# Pre-injury administration of morphine prevents development of neuropathic hyperalgesia through activation of descending monoaminergic mechanisms in the spinal cord in mice

**DOI:** 10.1186/1744-8069-1-19

**Published:** 2005-06-03

**Authors:** Md Harunor Rashid, Hiroshi Ueda

**Affiliations:** 1 Division of Molecular Pharmacology and Neuroscience, Nagasaki University Graduate School of Biomedical Sciences, Nagasaki 852-8521, Japan; 2 Dept of Integrative Physiology, Kyushu University Graduate School of Medical Sciences, 3-1-1 Maidashi, Higashi-ku, Fukuoka 812-8582, Japan

## Abstract

The present study examined whether pre-injury administration of morphine can prevent partial sciatic nerve injury-induced neuropathic pain in mice. We observed that pre-injury administration of subcutaneous (s.c.) and intracerebroventricular (i.c.v.) morphine dose-dependently prevented the development of both thermal and mechanical hyperalgesia at 7 days following nerve injury in mice. The pre-injury morphine (s.c.)-induced analgesia was significantly blocked by pretreatment with naloxone injected s.c. or i.c.v., but not i.t., suggesting that systemic morphine produced the pre-emptying effects mainly by acting at the supra-spinal sites. Since it is believed that activation of descending monoaminergic mechanisms in spinal cord largely contributes to the supra-spinal analgesic effects of morphine, we investigated the involvement of serotonergic and noradrenergic mechanisms in spinal cord in the pre-injury morphine-induced analgesic effects. We found that pre-injury s.c. morphine-induced analgesic effect was significantly blocked by i.t. pretreatment with serotonergic antagonist, methysergide and noradrenergic antagonist, phentolamine. In addition, pre-injury i.t. injection of serotonin uptake inhibitor, fluoxetine and α2-adrenergic agonist, clonidine significantly prevented the neuropathic hyperalgesia. We next examined whether pre-injury morphine prevented the expression of neuronal hyperactivity markers such as c-Fos and protein kinase C γ (PKCγ) in the spinal dorsal horn. We found that pre-injury administration of s.c. morphine prevented increased expressions of both c-Fos and PKCγ observed following nerve injury. Similar results were obtained with i.t. fluoxetine and clonidine. Altogether these results suggest that pre-injury administration of morphine might prevent the development of neuropathic pain through activation of descending monoaminergic pain inhibitory pathways.

## Background

One of the critical factors that initiate and maintain chronic pain is central sensitization where neurons in the spinal dorsal horn become more excitable due to prior repetitive noxious stimuli [1]. Thus, preventing the initial cascade of neural events may eliminate the long-term hypersensitivity. Initiating an analgesic regimen before onset of such noxious stimulus in an attempt to prevent the central sensitization is known as preemptive analgesia [2]. The concept of preemptive analgesia was originally proposed at the beginning of the last century by Crile [3]. Since the revival of the concept again by Woolf in 1983 in experimental animals [4], it has been practiced in the clinic in order to lessen post-operative pain following various surgical operations [2,5-8]. In spite of some controversies regarding the effectiveness of preemptive analgesia in some clinical settings, it may have tremendous economic benefits due to savings from reduced length of hospital stay, fewer post-operative complications, and improved quality of life [9]. Preemptive analgesia strategies mainly include infiltration with local anesthetics, nerve block, epidural block, use of analgesics such as morphine, NSAIDS, cyclooxygenase (COX)-2 inhibitors, inhibition of pain pathways by NMDA antagonists etc. [2,7-9].

Both clinical and preclinical studies suggest that pre-operative administration of morphine and other opioid analgesics can improve post-operative pain management [10-12]. Recent studies also demonstrate that opioids are able to prevent central sensitization in animal models of pain [13]. However, the effectiveness of pre-injury morphine to prevent induction of nerve injury-induced neuropathic pain has been largely unknown. Smith et al., [14] reported that pre-injury administration of systemic morphine was less effective than α2-adrenergic receptor agonist, clonidine in preventing the mechanical hyperalgesia in a rat model of mononeuropathy. On the other hand, Puke and Wiesenfeld-Hallin [15] showed that pre-operative intrathecal administration of morphine, but not clonidine, prevented the autotomy behavior in a rat peripheral axotomy model. Therefore the exact mechanism of preemptive analgesic effect of morphine in nerve injury-induced pain is yet to be clarified. It is well known that μ-opioid receptors (MOP) are largely distributed in different brain areas with some distribution in the spinal dorsal horn and dorsal root ganglion neurons [16]. The analgesic effect of systemic morphine is, however, mainly produced by activation of MOP in the periaqueductal grey (PAG), and brainstem nucleus raphe magnus (NRM) and locus coeruleous (LC), ultimately activating the descending pain inhibitory pathways consisting mainly of the noradrenergic and serotonergic neuronal terminals to the spinal cord [17]. Direct activation of the spinal MOP by intrathecal morphine is also reported to produce potent acute analgesia in experimental animals [18,19]. However, the efficacy of both systemic and spinal morphine is reduced in neuropathic pain [19,20]. Therefore, the concept of pre-operative application of morphine could provide a way out to circumvent the limitations associated with acute administration of morphine against such painful conditions.

In the present study, we utilized a systematic approach to see the exact contribution of supra-spinal and spinal μ-opioid receptors in the pre-injury morphine-induced analgesic effects by administering it through various routes. We also examined the contribution of spinal monoaminergic systems in the pre-injury morphine-induced analgesic effects. In addition, we observed the effects of pre-injury administration of morphine on nerve injury-induced increases in expression of c-Fos and PKCγ, two important markers of neuronal hyperactivity, in the spinal cord.

## Results

### Pre-, but not post-, injury administration of morphine prevented the development of thermal and mechanical hyperalgesia in nerve-injured mice

**Figure 1:**
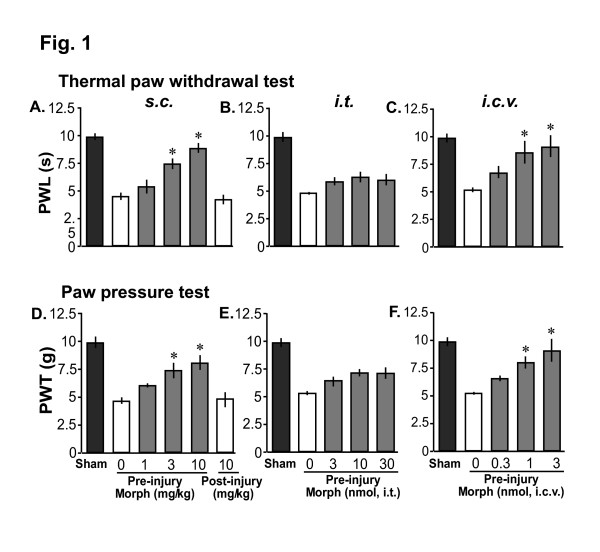
Pre-injury morphine-induced analgesia in partial sciatic nerve injury model mice. A-F: Pre-injury single administration of s.c. (1, 3, 10 mg/kg), and i.c.v. (0.3, 1, 3 nmol), but not i.t. (3, 10, 30 nmol), morphine prevented the development of thermal (A-C) and mechanical hyperalgesia (D-F) in the nerve-injured mice at 7 days following injury compared with vehicle pretreatment (Pre-injury Morph 0). Post-injury injection of 10 mg/kg s.c. morphine (30 min after surgery) failed to prevent the development of thermal or mechanical hyperalgesia. 'Sham' indicates thermal paw withdrawal latencies or mechanical paw withdrawal thresholds in control sham-operated mice. 'Morph' is morphine. Each data represents mean ± SE from 6–7 mice; *p < 0.05 compared with the group receiving vehicle as pretreatment.

Morphine, injected subcutaneously (s.c.) 30 min before partial sciatic nerve injury in mice, dose-dependently prevented the development of both thermal and mechanical hyperalgesia observed at 7 days after nerve injury with a significant effect at doses of 3 and 10 mg/kg s.c. (Fig. 1A,D). However, 10 mg/kg of s.c. morphine, injected 30 min after the nerve injury operation, failed to prevent the development of thermal or mechanical hyperalgesia in mice (Fig. 1A,D; last column). The pre-emptying effects of pre-injury morphine on post-injury pain continued at day 14 after nerve injury (data not shown). When we examined the effect of pre-injury intrathecal (i.t.) and intracerebroventricular (i.c.v.) morphine, only i.c.v. morphine significantly prevented the development of thermal and mechanical hyperalgesia (Fig. 1C,F). Although i.t. morphine produced some pre-emptying effects, it was statistically insignificant (Fig. 1B,E). Post-injury injection of both i.t. and i.c.v. morphine at 30 min after nerve injury did not produce any post-operative analgesia in the nerve-injured mice at 7 day after injury (data not shown). Pre-operative injection of morphine in sham-operated group of mice had no effect on the withdrawal latencies or thresholds observed at 7 days after sham operation (data not shown).

### Pre-injury subcutaneous (s.c.) morphine-induced analgesia was mediated by MOP in the supra-spinal sites

**Figure 2:**
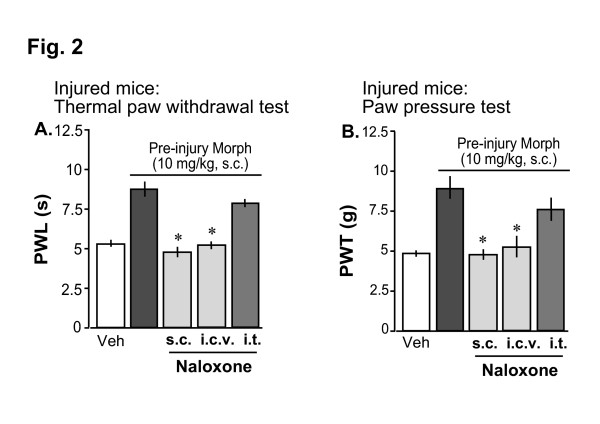
Involvement of supra-spinal MOPs in the pre-injury morphine-induced analgesic effects. A,B: Blockade of pre-injury morphine (10 mg/kg, s.c.)-induced analgesia by pretreatment with s.c. (1 mg/kg) or i.c.v. (1 nmol), but not i.t. (10 nmol), injection of the μ-opioid receptor antagonist naloxone in thermal paw withdrawal (A) and mechanical paw pressure (B) tests. Thermal paw withdrawal latencies (PWL) or mechanical paw withdrawal thresholds (PWT) were measured at 7 days following nerve injury. 'Morph' is morphine. Each data represents mean ± SE from 6–7 mice; *p < 0.05 compared with the vehicle pretreatment. 'Veh' indicates group of mice receiving only vehicle saline before nerve injury operation.

Since the above results suggest involvement of supra-spinal sites for pre-emptying effects of morphine, we further examined the contribution of μ-opioid receptors (MOP) in the brain and spinal cord in mediating the pre-injury systemic morphine-induced analgesic effect. As shown in Fig. 2A,B, pre-injury s.c. morphine (10 mg/kg)-induced analgesia was significantly blocked by pretreatment with 1 mg/kg of s.c. naloxone, a MOP antagonist. Moreover, pre-injury s.c. morphine-induced preemptive analgesia was blocked by pretreatment with 1 nmol of i.c.v. naloxone, but not with 10 nmol of i.t. naloxone, suggesting the involvement of supra-spinal MOPs in mediating the systemic morphine-induced pre-emptying effects. The s.c., i.t. and i.c.v. doses of naloxone were chosen from previous studies in mice [21].

### Pre-injury morphine-induced analgesia is mediated through activation of the descending monoaminergic pathways in the spinal cord

**Figure 3:**
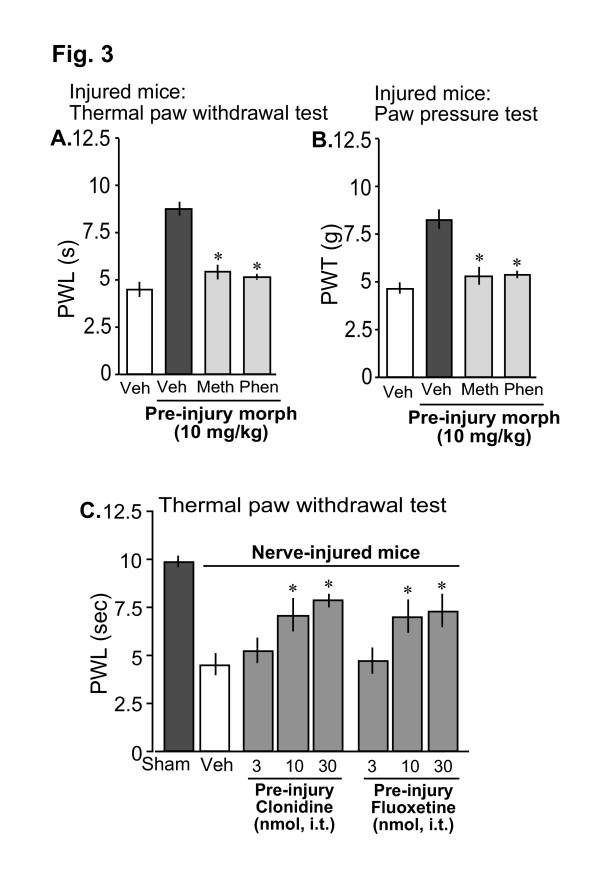
Involvement of descending monoaminergic pathways for the pre-injury morphine-induced analgesic effects. A,B: Pre-injury morphine (10 mg/kg. s.c.)-induced analgesia in thermal paw withdrawal (A) and mechanical paw pressure (B) tests were blocked by i.t. pretreatment with 3 nmol of serotonergic antagonist methysergide (Meth) or 10 nmol of adrenergic antagonist phentolamine (Phen). C: Dose-dependent pre-emptying effects induced by pre-injury i.t. injection of adrenergic agonist clonidine and serotonin uptake inhibitor fluoxetine in thermal paw withdrawal test. Thermal paw withdrawal latencies (PWL) or mechanical paw withdrawal thresholds (PWT) were measured 7 days following nerve injury. 'Morph' is morphine. Each data represents mean ± SE from 6–7 mice; *p < 0.05 compared with the group receiving vehicle as pretreatment.

Since the analgesic effect of systemic morphine is believed to be largely mediated through activation of the descending monoaminergic pain inhibitory pathways in the spinal cord [17], we next examined the effects of i.t. injections of antagonists of serotonergic and noradrenergic systems. As shown in Fig. 3A and 3B, i.t. pretreatment with 3 nmol of methysergide, a serotonergic antagonist, significantly blocked the pre-injury morphine-induced pre-emptying effects. Intrathecal (i.t.) pretreatment with 10 nmol of phentolamine, a noradrenergic antagonist also blocked the post-operative analgesia produced by pre-injury administration of 10 mg/kg of s.c. morphine. The antagonists alone had no effects in the nerve-injured mice (data not shown). We performed further experiments to explore whether i.t. injection of serotonergic or noradrenergic agonists could produce similar pre-emptying effects. As shown in Fig. 3C, i.t. pre-injury injection of serotonin uptake inhibitor fluoxetine and α-2 adrenergic agonist clonidine, dose-dependently prevented the thermal and mechanical hyperalgesia in injured mice. The doses of methysergide and phentolamine were chosen from previous reports in mice [21].

### Pre-injury administration of morphine prevented nerve injury-induced expression of c-fos in the spinal cord

**Figure 4:**
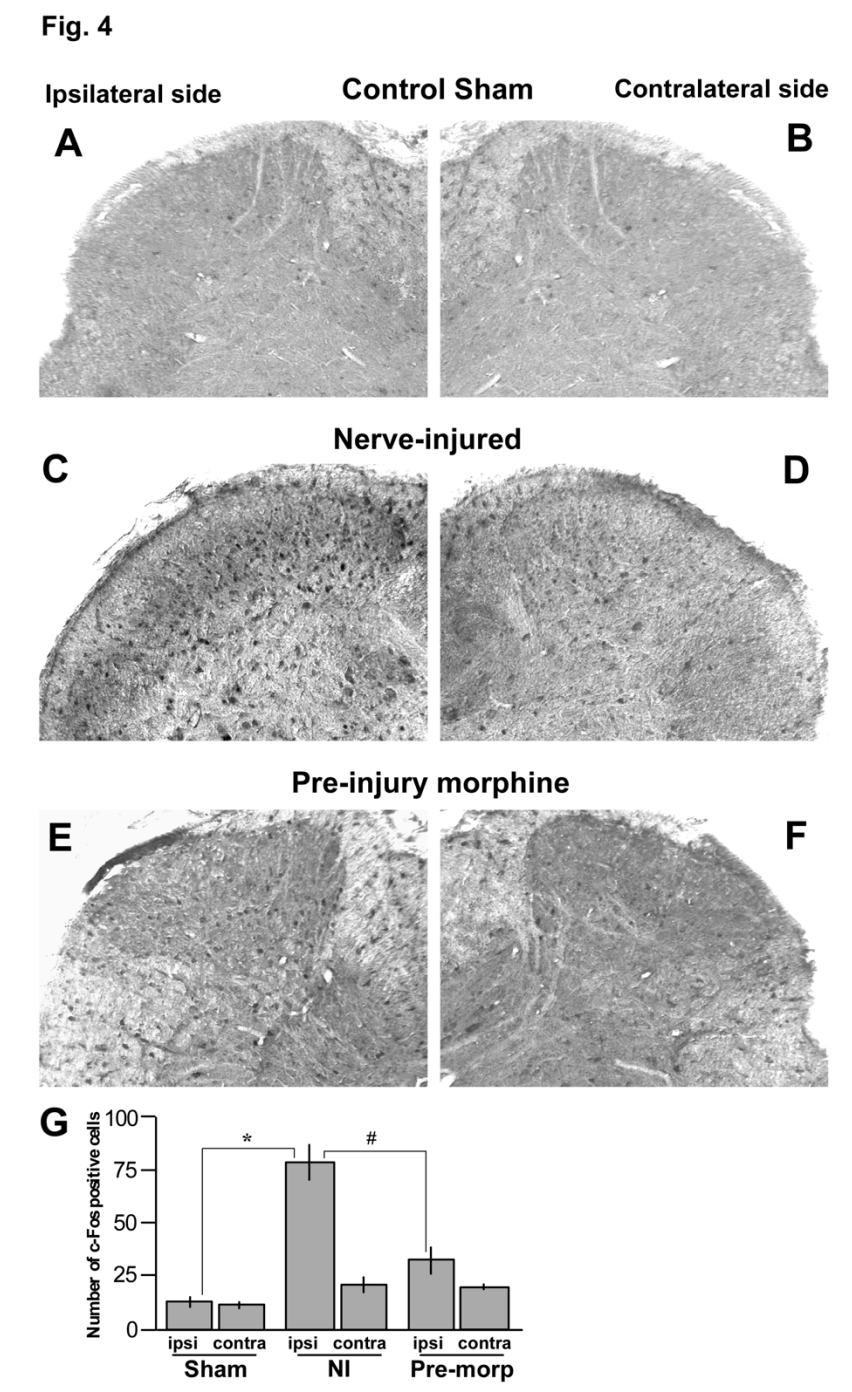
Prevention of nerve injury-induced expression of c-Fos protein in the spinal cord by pre-injury administration of morphine. A,B: Only a few c-Fos-positive neurons were found the spinal dorsal horn of control sham-operated mice. C: At 7 days following peripheral nerve injury, numerous c-Fos-positive cells were observed in most dorsal horn laminas of ipsilateral side of the spinal cord. D: Some c-Fos-positive cells were also observed in the contralateral side. E,F: Pre-injury administration of 10 mg/kg of s.c. morphine prevented the injury-induced expression of c-Fos in the spinal dorsal horn. G: Histogram of the number of c-Fos positive cells in ipsi- and contralateral sides of sham-operated (sham), nerve-injured (NI) and pre-injury morphine treated (Pre-morp) mice from three animals of each treatment group taking three sections from each animal. * p < 0.05 indicates statistically significant difference compared between the ipsilateral sides of sham and nerve-injured mice. # p < 0.05 indicates significant compared between the ipsilateral sides of the nerve-injured and pre-injury morphine treated mice. Scale bar represents 100 μm for all images.

The immediate early gene *c-fos *is an important marker of neuronal activity. Expression of Fos protein is rapidly increased in the spinal cord in response to peripheral noxious stimuli [22]. Induction of neuropathic pain has been correlated with nerve injury-induced short-term as well as long-term c-Fos expression in the spinal dorsal horn [23]. In the rat chronic constriction injury (CCI) model of neuropathic pain, c-Fos expression was increased in the spinal dorsal horn with a peak increase at 3 days after injury, and persisted for 30 days before returning to baseline level [23]. In the present report we examined whether pre-injury administration of morphine could prevent the c-Fos expression in the spinal cord following partial sciatic nerve ligation. As shown in Fig. 4C, partial sciatic nerve ligation increased expression of c-Fos in the ipsilateral side at 7 days after the injury compared with the ipsilateral side of the sham-operated mice (Fig. 4A). Some increase in c-Fos expression was also observed in the contralateral side of nerve-injured mice (Fig. 4D), compared with the control sham-operated mice (Fig. 4B). Pre-injury injection of 10 mg/kg of s.c. morphine 30 min before surgery prevented the injury-induced c-Fos expression in the spinal cord dorsal horn (Fig. 4E,F). When we counted the number of c-Fos-positive cells in the dorsal horn of the spinal cord of these groups of mice, we found that there was a significant increase in number of Fos-positive cells in the ipsilateral side of nerve-injured mice compared with the ipsilateral side of sham-operated mice at 7 days following injury (Fig. 4G). Pre-injury administration of 10 mg/kg of s.c. morphine significantly reduced the number of Fos-positive cells in the ipsilateral dorsal horn of nerve-injured mice (Fig. 4G).

### Pre-injury morphine, clonidine and fluoxetine prevented injury-induced increase in PKCγ expression in the spinal dorsal horn

**Figure 5:**
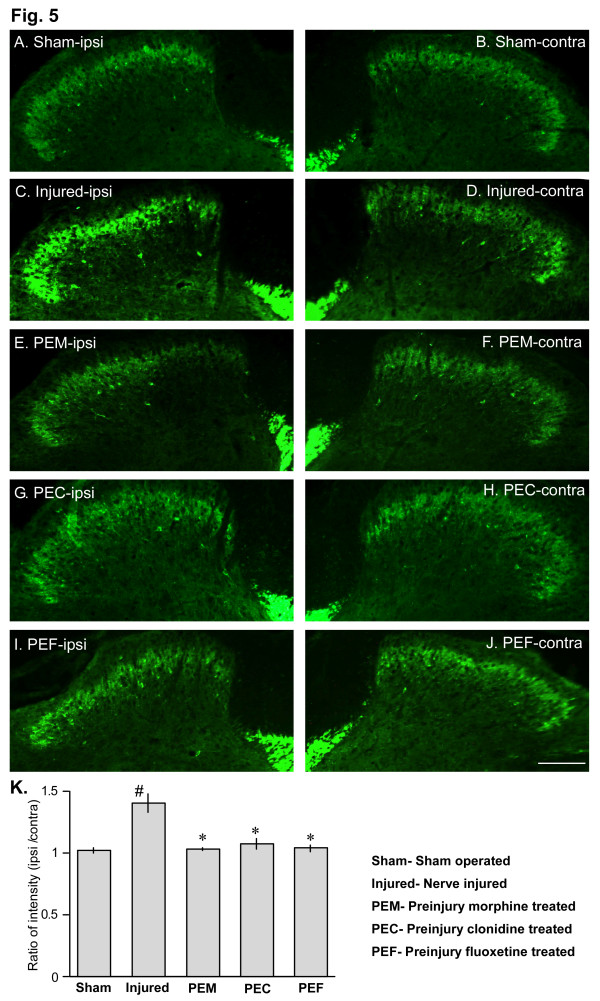
Prevention of nerve injury-induced increase in PKCγ expression in the spinal dorsal horn by pre-injury morphine. A,B: PKCγ immunoreactivity in the ipsilateral (A) and contralateral (B) sides of spinal dorsal horn of sham-operated mice. C,D: PKCγ-IR in the ipsilateral (C) and contralateral (D) sides of nerve-injured mice at 7 days after nerve injury. E,F: PKCγ-IR following pre-injury administration of morphine (10 mg/kg, s.c.) in ipsilateral (E) and contralateral (F) sides of nerve-injured mice at day 7. G-J: Pre-injury i.t. injection of clonidine (30 nmol) and fluoxetine (30 nmol) also prevented the injury-induced increase in PKCγ expression in spinal dorsal horn. K: Quantification of PKCγ immunoreactive fluorescence in ipsilateral sides of sham operated (sham), nerve-injured (injured), pre-injury morphine (PEM), pre-injury clonidine (PEC) and pre-injury fluoxetine (PEF) treated mice. Quantification of staining intensity was done using Scion imaging software for Macintosh. The data were represented as the ratio of staining intensity between ipsilateral and contralateral sides of each section in the treatment groups. Data were taken from three animals of each treatment group taking three separate sections from each animal. *, # p < 0.05. The scale bar represents 100 μm for all images.

We also examined the effects of pre-injury administration of morphine on expression of PKCγ in the spinal dorsal horn. Increased expression of PKCγ, an important component of central sensitization, is well documented in animal models of peripheral neuropathic pain [24,25]. In the present study we observed an increased expression of PKCγ in ipsilateral side of the spinal dorsal horn of partial sciatic nerve-injured mice at 7 days following injury compared with the ipsilateral side of control sham-operated mice (Fig. 5A–D,K). Pre-injury administration of 10 mg/kg of s.c. morphine almost completely prevented the increase in PKCγ expression observed following nerve injury (Fig 5E,F,K). Pre-injury i.t. administration of clonidine (30 nmol) and fluoxetine (30 nmol) also significantly prevented the injury-induced increase in PKCγ expression in spinal dorsal horn (Fig. 5G–J,K).

## Discussion

In the present study, we demonstrated that a pre-injury single administration of morphine could prevent development of thermal and mechanical hyperalgesia in the partial sciatic nerve injury model of neuropathic pain. We further demonstrated that pre-injury morphine-induced analgesia might be mediated through activation of the descending monoaminergic pathways in the spinal cord. In the present study, while pre-injury subcutaneous (s.c.) and intracerebroventricular (i.c.v.) morphine produced significant pre-emptying effects, intrathecal (i.t.) morphine only slightly prevented the injury-induced hyperalgesia which was statistically insignificant. These results suggest that the density of μ-opioid receptors (MOP) in the spinal cord might be insufficient to produce the necessary analgesic effect that could prevent the nerve injury-induced initial barrage of neuronal stimulation, ultimately leading to the development of central sensitization. Such results might be in contrary to the observation of strong acute analgesia by intrathecal morphine as reported previously [18,26]. However, we speculate that the distribution of MOP in the spinal cord is indeed sufficient to produce acute analgesia but might be insufficient to produce the necessary analgesia to prevent the nerve injury-induced initial barrage of neuronal stimulation compared with the supra-spinal sites, where MOPs are densely distributed [16]. Moreover, blockade of systemic morphine-induced preemptive analgesia by s.c. and i.c.v., but not i.t. naloxone further indicates the involvement of supra-spinal MOPs in the pre-emptying effects of morphine.

It is well known that the analgesic effect of systemic morphine is largely mediated by activation of MOPs in brainstem nuclei such as nucleus raphe magnus (NRM) and locus coeruleous (LC) that exert a net inhibitory effect on nociceptive transmission through descending monoaminergic pain inhibitory pathways in the spinal cord [17]. The serotoninergic and noradrenergic systems in the spinal cord also mediate the antinociception produced by intracerebroventricular (i.c.v.) injection of morphine [27]. Consistent with these lines of evidence, pre-injury systemic morphine-induced analgesia in our study was significantly blocked by intrathecal (i.t.) pretreatment with both serotonergic and noradrenergic antagonists. Moreover, pre-injury i.t. injection of the serotonin uptake inhibitor fluoxetine and the α-2 adrenergic agonist clonidine produced significant analgesia, further indicating ability of the spinal serotonergic and noradrenergic system to produce sufficient level of pre-emptying effects. It has been reported that both serotonergic and noradrenergic terminals innervate the presynaptic terminals of small nociceptive primary afferents [28], and can inhibit neurotransmitter release [29].

Pre-injury administration of morphine also prevented the injury-induced increases in expression of c-Fos and PKCγ in the spinal cord. Induction of neuropathic pain has been correlated with nerve injury-induced short-term as well as long-term c-Fos expression in the spinal dorsal horn [23]. The blockade of nerve injury-induced c-Fos expression in the spinal cord by pre-injury morphine indicates that preemptive systemic morphine is able to prevent injury-induced neuronal cascade that ultimately might cause neuropathic pain. Activation of PKC in the spinal cord dorsal horn, which triggers sustained activation of N-methyl-D-aspartate (NMDA) receptors, also serves as a marker of central sensitization [30]. Among different isoforms of PKC, the γ isoform was well studied with regard to neuropathic pain. Increased expression of PKCγ is well documented in animal models of peripheral neuropathic pain [24,25,31]. Reduced hyperalgesia was also observed following peripheral nerve injury in mice lacking PKCγ [32]. In the present study, we observed increased expression of PKCγ in the spinal dorsal horn following partial sciatic nerve injury, and pre-injury administration of morphine prevented such increased expression. It has been reported that majority of PKCγ-containing cells in the spinal dorsal horn are mainly excitatory interneurons, and does not contain the μ-opioid receptors (MOP) [33]. This might be also one of the reasons for the ineffectiveness of intrathecally injected morphine to produce significant preemptive analgesia in our studies. Finally, the blockade of injury-induced increase in PKCγ expression by i.t. clonidine and fluoxetine suggest that activation of descending monoaminergic system in spinal cord by systemic morphine might have prevented the development of central sensitization.

## Conclusion

In conclusion, results of the present study demonstrate that pre-injury administration of morphine could prevent the development of peripheral nerve injury-induced neuropathic pain through activation of descending pain inhibitory mechanisms. These results may improve management of chronic neuropathic pain by proper use of morphine.

## Methods

### Animals

Male ddY mice weighing 20–25 g were used throughout the experiments. The mice were housed in a room maintained at 21 ± 2°C, 55 ± 5 % relative humidity and an automatic 12-h light/dark cycle with free access to standard laboratory diet and tap water. The animals were adapted to the testing environment (maintained at 21 ± 2°C, 55 ± 5 % relative humidity and 12-h light/dark cycle) by keeping them in the testing room 24 h before the experiments. Experiments were performed during the light phase of the cycle (10:00 – 17:00). All procedures were approved by Nagasaki University Animal Care Committee and complied with the recommendations of International Association for the Study of Pain [34].

### Drugs and injection methods

Following drugs were purchased: morphine hydrochloride (Takeda Pharma. Co. Ltd., Japan), naloxone hydrochloride, methysergide maleate, phentolamine hydrochloride, fluoxetine hydrochloride, and clonidine hydrochloride (all from Sigma Co., St Louis, MO, USA). All drugs were dissolved in physiological saline. Physiological saline was used for control injections. The intrathecal (i.t.) injections were performed free hand between spinal L5 and L6 segments according to the method of Hylden and Wilcox [35]. The intracerebroventricular (i.c.v.) injections were carried out into the left lateral ventricle of mice. Injections were performed using a Hamilton microsyringe fitted with a 26-gauge i.c.v. needle, according to the method of Haley and McCormick [36]. The site of injection was 2 mm caudal and 2 mm lateral to the bregma, and 3 mm in depth from the skull surface. Both i.t. and i.c.v. injections were given in a volume of 5 μl. The mice received the subcutaneous (s.c.) injections in a volume of 0.1 ml/10 g body weight.

### Partial ligation of sciatic nerve

Partial ligation of the sciatic nerve of mice was performed under pentobarbital (50 mg/kg i.p.) anesthesia, following the methods of Malmberg and Basbaum [37]. Briefly, the common sciatic nerve of the right hind limb of mice was exposed at high thigh level through a small incision and dorsal 1/3 to 1/2 of the nerve thickness was tightly ligated with a silk suture. The wound was closed with a single muscle suture and antibiotic powder was dusted over the wound area following surgery. Sham operation was performed similarly except without touching the sciatic nerve. Immediately following surgery, the animals were kept in a soft bed cage with some food inside so that the animals could feed themselves without difficulty in standing. The wound healed within 1–2 days and the animals behaved normally. Experiments were carried out at 7 or 14 days post-ligation.

### Hargreaves thermal paw withdrawal test

Analgesia was measured from the latency to withdrawal evoked by exposing the right hind paw to a thermal stimulus. Mice were placed under Plexiglas cages on top of a glass sheet. The thermal stimulus (IITC Inc., Woodland Hills, CA, USA) was positioned under the glass sheet to focus the projection bulb exactly on the middle of plantar surface of the animals. A mirror attached to the stimulus permitted visualization of the undersurface of the paw. After one hour of adaptation, paw withdrawal latencies were measured at every 10 min interval until 60 min with vehicle or drug pretreatment. A cut-off thermal latency of 20 s was set in order to prevent tissue damage.

### Paw pressure test

Experiments were performed as described previously [38]. Briefly, mice were placed under a Plexiglas chamber on a 6 mm × 6 mm wire mesh grid floor and were allowed to accommodate for a period of one hour. The mechanical stimulus was then delivered onto the middle of the plantar surface of right hind-paw using a Transducer Indicator (Model 1601, IITC Inc., Woodland Hills, USA). The paw withdrawal thresholds were measured at every 10 min interval until 60 min with vehicle or drug pretreatment. In this experiment, a cut-off pressure of 20 g was set to avoid tissue damage.

### DAB immunostaining for c-Fos

Mice were deeply anesthetized with i.p. pentobarbital and perfused transcardially with 40 ml of 0.1 M potassium free phosphate buffered saline (K^+ ^free PBS, pH 7.4) followed by 40 ml of 4% paraformaldehyde (PFA) in 0.1 M K^+ ^free PBS. The spinal cord between L4 – L5 segments was removed and post-fixed in 4% PFA for 1 hour. Then, the sample was transferred to 25% sucrose solution (in 0.1 M K^+ ^free PBS) overnight for cryoprotection. Next day, the spinal cord sample was fast-frozen in cryoembedding compound on a mixture of ethanol and dry-ice and stored at -80°C until use. The spinal cord sample was cut as 20 μm thick transverse sections with a cryostat, thaw-mounted on silane-coated glass slide and air dried overnight at room temperature (RT). For c-Fos immunolabeling, spinal cord sections were washed 3 times with K^+ ^free PBS for 5 min each then incubated in excess 100% methanol with 0.1% H_2_O_2_. After 3 washings with K^+ ^free PBS, the sections were incubated in excess blocking buffer containing 10% normal goat serum and 2% bovine serum albumin in PBST (2% NaCl, 0.1% Triton-X 100 in K^+ ^free PBS) for 60 min at RT. The sections were washed and reacted with rabbit polyclonal antibody raised against the c-Fos protein (1:1000 in 2% BSA in PBST solution; *sc-7202*, Santa Cruz Biotechnology, CA, USA) at 4°C overnight. After thorough washings, the sections were incubated with secondary antibody, biotinylated anti-rabbit IgG (1:200 in 2% BSA in PBST solution; Vector, CA, USA) at RT for 60 min, and subsequently with ABC complex (Vector, CA) at RT for 60 min. The antigen-antibody reaction sites were visualized by incubation with a solution containing 0.005% 3,3'-diaminobenzidine tetrahydrochloride (DAB; Dojindo, Japan), 0.002% H_2_O_2_, 0.001% nickel ammonium sulfate and 0.002% cobalt chloride in 0.1 M K^+ ^free PBS until the black reaction products appear. The reaction was stopped by washing with ice-cold PBS. After 3–4 washings, the sections were cover-slipped and visualized under a light microscope. The number of c-Fos-positive cells in the ipsi-and contralateral sides of dorsal horn gray mater of the spinal cord was then counted from lamina I-VI and plotted in a bar graph.

### Fluorescence immunohistochemistry for PKCγ

The spinal cord sections were prepared as described above. For immunostaining of PKCγ, the spinal cord sections were first pre-blocked with blocking buffer containing 10% normal goat serum and 2% bovine serum albumin in PBST. The sections were then reacted with a rabbit polyclonal antibody raised against the γ isoform of protein kinase C (1:500 in 2% BSA in PBST solution; *sc-211*, Santa Cruz Biotechnology, CA, USA) at 4°C overnight. The sections were then incubated with a FITC-conjugated anti-rabbit IgG (1:200; Santa Cruz Biotechnology) for 60 min at RT. The sections were washed thoroughly, cover-slipped with Perma Fluor (Thermo Shandon, Pittsburgh, PA, USA) and examined under a fluorescence microscope (Olympus, Tokyo, Japan). Quantification of the intensity of PKCγ-positive fluorescence was then done using Scion imaging software for Macintosh (Scion Corporation, USA).

### Statistical analysis

Statistical evaluations of the data were performed by comparison with repeated measures analysis of variance (ANOVA) with suitable post-hoc tests. The criterion of significance was set at *p *< 0.05. All results are expressed as the mean ± SEM.

## List of Abbreviations

s.c., subcutaneous; i.t., intrathecal; i.c.v., intracerebroventricular; MOP, μ-opioid receptor; PBS, phosphate-buffered saline; AUC, area under the curve; ANOVA, analysis of variance; PKCγ, protein kinase C γ isoform.

## Competing interests

There are no financial as well as non-financial competitions with any other people or organizations.

## Authors' contributions

H. Ueda contributed to the conception and drafting of manuscript and has final approval of this version to be published.

M.H. Rashid contributed to the conception, design, data acquisition and drafting of the manuscript, and has final approval of this version to be published.
